# Biosafety Regulatory Reviews and Leeway to Operate: Case Studies From Sub-Sahara Africa

**DOI:** 10.3389/fpls.2020.00130

**Published:** 2020-03-06

**Authors:** John Komen, Leena Tripathi, Boniface Mkoko, Daniel Osei Ofosu, Herbert Oloka, Doris Wangari

**Affiliations:** ^1^ Komen Bioscience Consultancy, Haarlem, Netherlands; ^2^ International Institute for Tropical Agriculture (IITA), Plant Biotechnology, Nairobi, Kenya; ^3^ Program for Biosafety Systems, Lilongwe, Malawi; ^4^ Biotechnology and Nuclear Agriculture Research Institute, Ghana Atomic Energy Commission, Accra, Ghana; ^5^ Program for Biosafety Systems, Kampala, Uganda; ^6^ Program for Biosafety Systems, Nairobi, Kenya

**Keywords:** policy & institutional actions, biotechnology, biosafety analysis, regulation, genome editing

## Abstract

While modern biotechnology and, specifically, genetic modification are subject of debate in many parts of the world, an increasing number of countries in Sub-Sahara Africa are making important strides towards authorizing general releases of genetically modified (GM) crop varieties for use by farmers and agribusinesses. Obviously, the documented economic and environmental benefits from planting GM crops—based on a track record of over two decades—are a major driver in the decision-making process. Another key factor is the increasing alignment of biosafety regulatory policies with progressive agricultural and rural development policies in Africa, resulting in—compared to past experiences—greater emphasis on anticipated benefits rather than risks in biosafety regulatory reviews. In several cases, this has led to expedited reviews of GM crop release applications, either for confined field trials or general environmental release, taking experiences and data from other countries into account. Such regulatory approaches hold promise as the pipeline of relevant, pro-poor GM crop applications is expanding as are the opportunities provided by novel plant breeding techniques. This review article analyses the shifting policy context in select African economies, resulting in adoption of new agricultural technology, and novel regulatory approaches used in biosafety decision-making. Case studies will be presented for Ghana, Kenya, Malawi, Nigeria and Uganda to analyze challenges, distill lessons learned and to present general policy recommendations for emerging economies.

## Context and Methodology: Regulation–Technology Interactions

As is the case generally when new technologies are introduced in society, there have been strong claims about the benefits and perceived adverse effects of agricultural biotechnology, specifically regarding genetically modified (GM) crops, and more recently regarding emerging techniques in plant breeding such as genome editing. Early concerns regarding genetic modification stimulated, from the 1980s onward, the creation of regulatory systems for food and feed safety and environmental risk assessment. In Africa, governments generally started the development of their national biosafety systems more recently and, as with other areas of safety regulation, the task has been difficult in terms of defining science-based regulations and enforcement. For most of them the Cartagena Protocol on Biosafety has set the starting point, as adopted in 2003 as a supplement to the Convention on Biological Diversity (CBD), and which seeks to address environmental impact from transboundary movement, management and safe use of genetically modified organisms (GMOs).

The adoption of regulatory policies for biosafety and food/feed safety should be seen as part of a broader emerging international regime increasingly affecting the access and use of genetic resources for food and agriculture. This international regime affects scientific leeway and freedom-to-operate in a major way: The development and deployment of new agricultural technologies gets increasingly regulated, and often impeded, as access to essential research inputs (such as genetic resources, or protected technology) and the release of research outputs such as new crop varieties, is slowed down or halted by overly restrictive regulations. This phenomenon is described in further detail in Komen (2012). Evidently, the existence of sovereign rights over a country's natural resources, including genetic resources, is now well established in international law. Traditionally, genetic resources for agriculture were considered a common heritage of humankind and generally there was free transnational flow and access to all biological materials wherever they were located. Over time this situation was perceived to cause asymmetry between countries with rich genetic resources, usually free providers of these resources, and countries without extensive biological resources but who used them in R&D and protecting research results as intellectual property. The CBD, from which the Cartagena Protocol on Biosafety originated, changed this concept and reinforced sovereign rights to States over their biological resources through “access and benefit sharing” (ABS) regimes. Rourke (2018) analyses the increasing legal obstacles to accessing genetic resources and concludes that:

*“The culmination of these barriers renders some biological research untenable and can result in the abandonment of research projects before they even commence.”* (Rourke, 2018)

The CBD's Cartagena Protocol on Biosafety, originally aimed at maximizing benefits of biotechnology for biodiversity conservation while minimizing adverse effects, tends to add to the complexity by emphasizing the “precautionary principle” in biosafety decision making, while providing only limited guidance on what constitutes a functional national regulatory framework. Therefore, it is the responsibility of national governments to come up with science-based and efficient biosafety policies. This is becoming critical as new agricultural technologies are emerging, such as those related to genome editing, for which new regulatory approaches and instruments may be required.

According to Wiener (2004), technology and regulation are generally regarded as adversaries, with regulation seen as inhibiting technological change. Particularly regarding GMOs, calls for precautionary regulation have been frequent and reflected in the Cartagena Protocol as well as in several national and sub-regional regulatory frameworks. In the same essay, Wiener (2004) emphasizes that technological change impacts regulation as well. For example, by introducing improved technologies and products, risk can be reduced leading to decreasing need for regulatory oversight. In addition, it is often argued that regulation may encourage innovation^1^ and competitiveness by promoting the introduction of cleaner and more cost-effective technologies. In cases where the chosen regulatory instrument is appropriate and well designed, technological change will progress. A prerequisite, according to Wiener, is the presence of “policy entrepreneurs” or “policy innovators” who: *“[W]ill develop and test new forms and approaches to regulation for greater effectiveness, les cost, less caustic side effects, and other describable attributes.”* Wiener's arguments are reaffirmed in OECD analyses on “regulatory reform”, defined as “*changes that improve regulatory quality, that is, enhance the performance, cost-effectiveness, or legal quality of regulations and related government formalities.”* (OECD, 1997).

While the present article does not aim at providing an academic analysis of biosafety regulation and regulatory reforms, it is important to point to emerging regulatory reforms, and factors driving those reforms, in several countries in sub-Sahara Africa. First and foremost, recent reforms reflect a growing body of literature regarding the actual benefits and adverse impacts of GM crops. Over time, as actual experience continues to grow in planting, processing and consuming GM crops, much clearer analyses emerge regarding their real impacts, which is in turn affecting regulatory approaches. For example, a recent study by the US National Academies of Science (NAS, 2016) undertook a thorough review of available primary literature. The study committee found little evidence to connect GM crops and their associated technologies with adverse agronomic or environmental problems. In addition, the committee also found that—while impacts differed greatly across different contexts—systematic reviews and formal meta-analyses of the performance of GM crops have consistently shown the following impacts:

- Reductions in yield damage by insects;
- Reductions in insecticide applications for target insect pests, resulting in substantial environmental and health benefits;
- Decreases in management time and increases in flexibility related to herbicide-resistant (HR) crops; and,
- Increases in gross (in some cases net) margins due to the adoption of GM crops, or combinations of all the above.

(NAS, 2016)

More specifically, Qaim (2019) concludes that, based on an in-depth analysis of available literature: *“Over the last 20 years, a large number of studies have been conducted, analyzing the effects of GM crop adoption on yield, pesticide use, farm profits, and other outcomes in different parts of the world. A meta-analysis has evaluated these existing studies, finding that GM crop adoption benefits farmers in most situations (…). On average, GM technology has increased crop yields by 22% and reduced chemical pesticide use by 37% (…). GM seeds are usually more expensive than conventional seeds, but the additional seed costs are compensated through savings in chemical pest control and higher revenues from crop sales. Average profit gains for adopting farmers are 68%.* (Qaim, 2019)

Analysis such as published by NAS (2016) increasingly play a role in biosafety decision-making as regulators become better able to weigh risks against benefits. This article takes a country case-study approach to further explore this development, using experiences from select countries in Africa. We will investigate recent progress and lessons learned, drawing recommendations for future policy reforms.

## Evolving Policy Context for Agricultural Biotechnology in Sub-Sahara Africa

Recognizing the important (potential) benefits of biotechnology to improving food security and rural development, governments across Africa have taken steps to establish an enabling policy framework to support adoption of biotechnology including GM crops and derived products. A more detailed analysis of relevant policies and regulations is presented below. Examples of recent policy decisions regarding GM crops include:

- Approvals for general release and commercial variety registration for insect-resistant, GM cotton hybrids in Ethiopia (2018), Kenya (2019), Malawi (2019) and Nigeria (2018). While farmers in Ethiopia started planting GM cotton at limited scale in 2019, GM seed distribution in Kenya, Nigeria and Malawi will start in 2020;
- Approval for general release and submission for variety registration for insect-resistant, GM cowpea in Nigeria (2018). Next step in this process will involve the registration, by the National Variety Release Committee, of GM cowpea as a new commercial variety followed by seed distribution by local companies.

In addition, with countries such as Ghana, Kenya and Uganda moving steadily from confined field trials (CFTs) towards general release applications, the setting for GM crop production in sub-Sahara Africa is rapidly changing. Until recently, only South Africa, Sudan and Burkina Faso had approved commercial production of GM crops. Right now, the regulatory pipeline is expanding and diversifying as illustrated by **Table 1**.

**Table 1:** Biotechnology crop pipeline in Ghana, Malawi, Nigeria and Uganda (active projects, 2019).

Product	Trait	Developer	Collaborating Institutes	Regulatory Status (year of approval)
**GHANA**				
Rice	Nitrogen use efficiency/water use efficiency/salt tolerance	Arcadia Biosciences	CSIR^1^—Crops Research Institute	CFT^2^ (2013)
Cowpea	*Maruca* podborer resistance	CSIRO^3^, AATF^4^	CSIR—Savannah Agricultural Research Institute	CFT (2013)
**KENYA**				
Maize	Drought tolerance	Bayer Crop Science	KALRO^5^, AATF, CIMMYT^6^	Approved for NPT (2016)
	Drought tolerance, insect resistance	Bayer Crop Science	KALRO, AATF, CIMMYT	CFT (2012)
	Stacked event of insect resistance and drought tolerance	Bayer Crop Science	KALRO, AATF, CIMMYT	CFT (2015)
Cotton	Insect resistance	Mahyco	KALRO	NPT (2016)
Sorghum	Biofortified sorghum with enhanced Vit A, Iron and Zinc	Pioneer Hi-Bred	KALRO, Africa Harvest	CFT (2015)
Cassava	Virus resistance (cassava brown streak disease)	DDPSC^7^	KALRO	CFT (2013)
	Virus resistance (African Cassava Mosaic Virus (ACMV) and Cassava Brown Streak Virus	MMUST^8^		CFT (2014)
Sweet potato	siRNA to sweet potato virus disease resistance	DDPSC	KALRO	CFT (2014)
**MALAWI**				
Cotton	Insect resistance	Mahyco	DARS^9^	Commercial varieties registered (2019)
Cowpea	*Maruca* pod borer resistance	CSIRO, AATF	LUANAR^10^	CFT (2015)
Banana	Bunchy top virus resistance	QUT^11^	DARS	CFT (2016)
Plantain	Bunchy top virus resistance	QUT	DARS	CFT (2018)
**NIGERIA**				
Cassava	Virus resistance; improved nutritional quality	DDPSC	NRCRI^12^	CFT approved (2019)
	Improved shelflife	IITA^13^		CFT (2018)
Cotton	Insect resistance	Mahyco	IAR^14^	Commercial varieties registered (2018)
Cowpea	Maruca pod borer resistance	CSIRO, AATF	IAR	General release approved (2018); submitted for variety registration (2019)
Rice	Nitrogen use efficiency/water use efficiency/salt tolerance	Arcadia Biosciences, CIAT, AATF	NCRI^15^	CFT (2014)
Soybean	Herbicide tolerance	MSU^16^	NABDA, NCRI	CFT approved (2019)
**UGANDA**				
Banana	Disease resistance	AATF, IITA	NARO^17^	Multi-location CFTs (2010)
	Nematode resistance		NARO	CFT (2012)
	Improved nutritional quality	QUT	NARO	CFT (2011)
Cassava	Virus resistance	DDPSC	NARO	Multi-location CFTs (2010)
Maize	Drought tolerance/insect resistance	Bayer Crop Science, AATF	NARO	Multi-location CFTs (2015)
Potato	Fungal resistance	CIP^18^	NARO	Multi-location CFTs (2015)
Rice	Nitrogen use efficiency/water use efficiency/salt tolerance	Arcadia Biosciences, CIAT, AATF	NARO	CFT (2012)
Soybean	Herbicide tolerance	MSU	NARO	Contained research (2016)

^1^CSIR, Council for Scientific and Industrial Research.

^2^CFT, Confined Field Trial.

^3^CSIRO, Commonwealth Scientific and Industrial Research Organisation, Australia.

^4^AATF, African Agricultural Technology Foundation.

^5^KALRO, Kenya Agricultural and Livestock Research Organisation.

^6^CIMMYT, International Maize and Wheat Research Center.

^7^DDPSC, Donald Danforth Plant Science Center, USA.

^8^MMUST, Masinde Muliro University of Science and Technology.

^9^DARS, Department of Agricultural Research Services.

^10^LUANAR, Lilongwe University of Agriculture and Natural Resources.

^11^QUT, Queensland University of Technology, Australia.

^12^NRCRI, National Root Crops Research Institute.

^13^IITA, International Institute for Tropical Agriculture.

^14^IAR, Institute for Agricultural Research.

^15^NCRI, National Cereals Research Institute.

^16^MSU, Michigan State University.

^17^NARO, National Agricultural Research Organisation.

^18^CIP, International Potato Center.

This increasing emphasis on agricultural biotechnology as a critical element in agricultural development policies is an important factor driving the expanding GM crop pipeline in Africa. Illustrative examples of such policies are presented below.

### Agricultural Policies Increasingly Supportive of Innovation

As noted in a recent analysis by AGRA, the Alliance for a Green Revolution in Africa (2018), agriculture is key to Africa's future considering that the continent has most of the world's arable land, over half of the African population is employed in the sector, and it is the largest contributor to total gross domestic product (GDP). Yet, Africa is still producing too little food and agricultural value-added products (AGRA, 2018). Productivity in the agricultural sector has been broadly stagnant since the 1980s. Similarly, government investments in agricultural R&D show a slightly declining trend. However, recent successes in achieving rapid agricultural growth (e.g., in Ethiopia) have encouraged governments to adopt much more growth-orientated policies, which are highlighted in this section.

Ghana's government in 2017 launched an ambitious initiative to industrialize Ghana with the establishment of agro-processing factories in each of the 216 districts in the country. This initiative dubbed “one-district-one-factory” is to be implemented through the private sector. The factories are expected to utilize raw materials readily available in the district where the factory is located. This program, coupled with another government initiative, “Planting for Food and Jobs”, is expected to boost agricultural production. This initiative has as its core: *“[T]he drive to motivate farmers to adopt improved, certified seeds and fertilizers through a private-sector marketing framework, by raising incentives and complimentary service provisions on the usage of inputs, good agronomic practices, marketing of outputs.”* (MOFA, 2017). While implementation of such programs may be slower than anticipated, together with supportive policies aimed at a more market-orientated agricultural sector, they provide clear guidance to technology developers testing and planning the release of GM crops in Ghana.

Kenya's “Vision 2030”, an overarching development policy aimed at becoming a middle-income country, focuses on agriculture as a key sector, which should drive the economy to an annual growth rate of around 10%. Agricultural policy in Kenya prioritizes a sharp increase in productivity and income growth, especially for smallholder farmers. More recently, the country's President's “Big 4 Agenda” emphasizes food security as the number one priority. This includes, among other elements, enhancing availability of basic staples such as maize, rice and potatoes, supporting agro-processing enterprises and enhancing large-scale crop production including cotton as an industrial crop. This Agenda acted as a boost to accelerating the introduction of GM, insect-resistant cotton. Revival of cotton production and local processing is among the Big 4 Agenda priorities and includes the large-scale planting of GM cotton to boost productivity.

For the past few decades, Malawi, like many other countries has been a net importer of food. Considering the challenge to become more self-sufficient and the important role agriculture plays in national development, the government of Malawi defined a Growth and Development Strategy (MGDS) in order to enhance agricultural growth, alleviate poverty and improve quality of life (Government of Malawi, 2017). The MGDS-III is a medium-term strategy designed to contribute to the country's long-term development goals. The current strategy covers a period of five years, from 2017 to 2022, with the objective to move Malawi to a productive, competitive and resilient nation primarily through sustainable agriculture while addressing water, climate change, and environmental management and population challenges. In the implementation plan/operation matrix, under agriculture sector, the Strategy identifies commercial application of agricultural biotechnologies as one of the priority activities which can contribute to increased agricultural production and productivity. In line with the MGDS, Malawi has secured a loan from the World Bank to transform Malawi's agricultural productivity through irrigation for period of 2019 to 2022. The objective of the project is to pull people out of poverty raising income levels of beneficiaries by overcoming the main production challenges of droughts and pests. These government initiatives indicate strong positive political will, which is critical to the sound regulation and adoption of any technologies including GM crops.

Nigeria's Agriculture Promotion Policy (2016–2020) document, titled, *“The Green Alternative” aims to “build an agribusiness economy capable of delivering sustained prosperity by meeting domestic food security goals, generating exports, and supporting sustainable income and job growth”* (FMARD, 2016). As the policy emphasizes, among other priorities, the need for productivity enhancements and innovation in Nigerian agriculture, and securing private sector investments, it has encouraged recent decisions to authorize the commercial release of GM crops such as insect-protected cotton and cowpea.

Agricultural policies in Uganda have been supportive towards exploiting the potential of GM crops since the development of the Plan for Modernization of Agriculture (PMA), some 20 years ago. The current overarching policy instrument, the Agriculture Sector Strategic Plan (ASSP) recognizes the need to enhance sustainable agricultural productivity and value addition using well-coordinated technological and service interventions (MAAIF, 2015). This policy further identifies the need to develop and implement a specific policy and regulatory framework for biotechnology in agriculture. In addition, the National Agricultural Research Act, 2005 was formulated with a key objective of transforming agricultural production into a modern science-based market-oriented system that is efficient, sustainable and profitable. The National Biotechnology and Biosafety Policy (2008) further strengthens the Government's commitment to utilize modern biotechnology tools for national transformation. These policy statements continue to guide GM research at the National Agricultural Research Organisation (NARO) in priority commodities such as maize, banana, and potato in line with the country's overall development policy statement, the Vision 2040, that also articulates the various sectors where biotechnology is seen as a strategic tool. These include agriculture, healthcare, industrial development, and environmental management.

### Functional Regulatory Frameworks Support Technological Change

In addition to the overall supportive policy initiatives sketched above, it is important to note that these countries have invested in establishing functional regulatory frameworks for GM crops, allowing decision makers to weigh potential benefits against potential adverse effects on the environment and human or animal health. A science-based and practical regulatory framework has become an important enabling factor for countries researching and adopting GM crops. **Table 2** provides an overview of regulatory frameworks for countries covered in this article.

**Table 2:** Biosafety regulatory frameworks in Ghana, Kenya, Malawi, Nigeria and Uganda (2019).

Cartagena Protocol Status (year ratified)	National Biosafety Law (year passed)	National Competent Authority	Subsidiary Regulations, Guidelines (year adopted)	Scientific Advisory Body
**GHANA**				
Ratified (2003)	Biosafety Act (2011)	National Biosafety Authority (NBA)	–Biosafety implementing regulations (2019) –General release guidelines (2016) –Guidelines for handling requests (2016)	Technical Advisory Committee (TAC)
**KENYA**				
Ratified (2003)	Biosafety Act (2009)	National Biosafety Authority (NBA)	–Contained use regulations (2011) –Environmental release regulations (2011) –Import, export and transit regulations (2011) –Labeling regulations (2012)	Board of Directors
**MALAWI**				
Ratified (2009)	Biosafety Act (2002)	Environmental Affairs Department (EAD)	Biosafety (Management of Genetically Modified Organisms) Regulations (2007)	National Biosafety Regulatory Committee (NBRC)
**NIGERIA**				
Ratified (2003)	National Biosafety Management Agency Act (2015)	National Biosafety Management Agency (NBMA)	National Biosafety Regulations (2017)	–National Biosafety Committee (NBC) –National Biosafety Technical Committee (NBTC)
**UGANDA**				
Ratified (2001)	Biosafety law pending Presidential assent; field trials regulated under Uganda National Council for Science and Technology Act (1990)	Uganda National Council for Science and Technology (UNCST)	–National Guidelines for Field Trials of Genetically Engineered Plants (2011) –National Guidelines for Containment (2007)	National Biosafety Committee (NBC)

In countries that are selected as case studies for this article, there has been progress in recent years in establishing functional national biosafety frameworks and growing expertise in GMO decision-making. An overview of key legal instruments and institutional setups is provided in **Table 2**. Generally, while it is very well possible to use existing legislation to regulate biotechnology and GMOs, countries have opted to develop new biosafety laws and centralized decision-making bodies – such as national biosafety authorities. This has proven to be an effective approach in most cases, while responding to the need for clear legal authority, as evidenced by the number and range of regulatory decisions made in recent years (presented in **Table 1**).

Clear legal authority through comprehensive biosafety laws has still provided regulatory agencies with options to adopt flexible and innovative approaches to GM decision making. This has, for example, included decisions to (i) accept field trial data from neighbouring countries to expedite reviews of applications for confined field trials (CFTs); (ii) accept data from local GM CFTs and multi-location trials to shorten procedures for variety registration trials; (iii) accept food/feed safety dossiers and assessments from trading partners for accelerated safety decision-making. The following examples serve to illustrate these points.

- In Ghana, the National Biosafety Committee (NBC) approved multi-locational trials (MLTs) for insect-resistant GM cotton in 2012 after accepting data from confined field trials (CFTs) conducted previously in Burkina Faso. Considering the similar agro-ecological zones for cotton production in the two countries, the National Biosafety Committee (NBC) decided to grant an exemption for local CFTs and move to MLTs right away. These trials were put on hold in 2016 due to Burkina Faso's decision to terminate the commercial registration of GM cotton.
- Nigeria adopted the same principle—accepting data from other countries—and authorized country-wide MLTs for GM cotton prior to endorsing its general release and commercial variety registration in 2018.
- Kenya adopted fast-tracked protocols for variety registration trials involving GM crops that have gone through CFTs and MLTs, shortening the time required for DUS/VCU^2^ performance trials from two planting seasons to one.

These examples confirm that, increasingly, governments in Africa can adjust their regulatory decision-making processes based on accrued scientific evidence.

### Political Challenges Remain

While the above sketched progress in regulatory decision-making is encouraging, it is important to note that, still, most African countries are only slowly progressing in implementing functional regulatory frameworks. And, even in countries where recent progress has been achieved, there is considerable potential for backsliding. Many governments experience political opposition to GM crops and modern agriculture generally, and proposed biosafety legislation and regulations are conveniently associated with “opening the gateways” for the introduction of GMOs. Political opposition is in most cases fuelled by anti-GM activism, which has slowed down or halted the adoption of biosafety legislation (see, for example, Afedraru, 2019). For a continent that could benefit greatly from improved planting material including GM crops, this progress is slow.

As analysed in detail by Komen and Koch (2017), despite significant effort and donor-agency resources devoted to biosafety capacity development, and despite progress in some countries such as those presented above, many countries still do not have adequate capacity to design and implement biosafety regulations. This remains a significant barrier to the testing and adoption of new crop varieties, including those developed by genome editing and other plant breeding innovations, which would open new opportunities to grow more food, enhance incomes and reduce environmental impact of agriculture. An uncertain regulatory environment discourages private and public sector investment into development of the pro-poor crops and traits that farmers need the most.

While many donor-funded support programmes have attempted to build national capacity for the regulation of GM crops, progress is uneven at best. An early analysis of this situation was presented by Johnston et al. (2008) and is still very relevant. Their report confirms that generally, countries with existing capacity for biotechnology R&D, that already receive applications for activities with GMOs, and that have high-level political support for biotechnology and biosafety capacity building, have made most advances and have benefited most from technical assistance (Johnston et al., 2008). This assessment found that a majority of developing countries, including most countries of Africa, Central Asia, Oceania and the Caribbean, were not yet able to manage modern biotechnology and implement their national biosafety frameworks. This general situation was confirmed in an independent evaluation commissioned by the CBD Secretariat in 2012 (CBD, 2012), and only slow progress has been made since then – with notable exceptions as indicated in this article.

For countries making progress despite the general challenges sketched above, important obstacles often occur at advanced stages of the regulatory process, when general release applications and commercial variety registration decisions are considered. In such cases, again based on analysis from countries that are focus for this article, the following hurdles occurred:

1. Lack of inter-ministerial collaboration and harmonization: As GM crops approach general release or market authorizations, government agencies become involved with responsibility for, e.g., food/feed safety, variety registration or, in some cases, for Environmental Impact Assessment (EIA). This has led to delays, inconclusive “conditional” release decisions and sometimes standstills in cases where these agencies lack familiarity with the biosafety regulatory review processes and tend to repeat safety reviews that were already completed. Early investment in establishing a coordinated, multi-agency framework is essential, coupled with policy consultations regarding harmonization of legal mandates.
2. Post-release requirements: Towards the final stages of the regulatory review process, post-release requirements are being considered related to, among other things, product labeling, product liability, co-existence, monitoring and surveillance. These concepts are in many cases not yet implemented and tested in African countries, associated expertise is low, and enforcement will be problematic. In the early phases of regulatory framework definition in Africa, strict post-release requirements were usually formulated and only later it is realized that these will form an impractical impediment to technology deployment.
3. High-level political will wavers: Finally, in the final stages of the decision-making process, government authorities get hesitant to fully authorize commercial cultivation of a GM crop particularly when this involves a GM food crop. The expected political/electoral consequences of such decisions, including the potential for public controversy, often affect such decisions. It also affects the decision-making regarding required policy reforms, as seen in the cases of (i) the continuation of a *de facto* GMO import ban in Kenya (Mukonyo, 2019), and (ii) the refusal by the President of Uganda to assent to a biosafety act that was passed twice by the Parliament of Uganda (Afedraru, 2019).

These hurdles have resulted in the current emphasis on primarily conducting CFTs with only slow progress towards general release and commercial cultivation, except for a non-food crop such as GM cotton. With the recent decision in Nigeria to authorize general release of GM cowpea, and its imminent registration as a commercial variety, this situation might change in the near future.

## Plant Science Moving Ahead: Application of Genome Editing for Improvement of Staple Crops in Africa—The Case of Banana and Cassava

While the R&D pipeline in Africa and, worldwide, adoption of GM crops steadily grows—in 2018, a total of 192 million hectares were planted with GM crops in 26 countries (ISAAA, 2018)—the emerging “new breeding techniques” (NBTs) are the latest addition to the plant breeder's toolbox as they offer the possibility of making genetic changes more precisely by targeting them to specific sites in the genome^3^. Especially the new tools for genome editing, like ODM (oligonucleotide mutagenesis) or CRISPR/Cas9 provide mechanisms to not just randomly increase genetic variation, as done by radiation or chemical mutagenesis, but also to precisely introduce mutations in genes of known functions to either impair or improve their function. NBTs have the potential to reduce the cost and time of bringing new products to the market since, compared with conventional breeding techniques, they can reduce the number of unwanted traits that might be co-transferred during the breeding process and that subsequently need to be removed. The greatest potential advantages of NBTs are their relative ease, precision, speed, and low cost, allowing breeders to focus more on the local growing conditions and to react more quickly to the changing needs and wants of growers and consumers.

With specific reference to sub-Sahara Africa, this precise genome editing has potential to revolutionize crop improvement. Notably, CRISPR/Cas9 has emerged as a powerful genome editing tool that can be used efﬁciently to induce targeted mutations in the genomes of plants species to produce improved varieties. CRISPR/Cas9 technology has been successfully applied in many organisms, including several plant species (Scheben et al., 2017). It has not only been established for model plants like *Arabidopsis* and *Nicotiana banthemiana* but also for complex crops like rice, wheat, maize, sorghum, tomato, soybean, apple, citrus, poplar, coffee (Ricroch et al., 2017; Breitler et al., 2018).

As in previous episodes of rapid changes in agricultural technology, the international centers that are part of the Consultative Group for International Agricultural Research (CGIAR) are at the forefront of incorporating NBTs in their research portfolio, in collaboration with national research organizations in sub-Sahara Africa. As a case in point, the International Center for Improvement of Maize and Wheat (CIMMYT, its acronym in Spanish), the Kenya Agriculture and Livestock Research Organisation (KALRO) and Corteva (formerly, DuPont Pioneer) have joined hands to exploit the gene editing (CRISPR-Cas) technology to improve maize and wheat germplasm. A specific example where this technology will be employed is to address maize lethal necrosis (MLN), a devastating viral disease that has spread rapidly in many countries of East Africa since it was first detected in Kenya. CIMMYT identified a strong source of resistance against MLN, have fine-mapped it to a 1 MB region of chromosome 6, and expect to isolate the gene that confers resistance. Among the first targets for gene editing will be the parents of long-standing commercial hybrids in East Africa that were developed before the appearance of MLN and have since become susceptible to the disease. The International Institute of Tropical Agriculture (IITA), one of the CGIAR is on the forefront of applying NBT for improvement of banana for developing resistance to diseases (Tripathi L. et al., 2019; Tripathi J.N. et al., 2019; Maxmen, 2019). Details are provided below.

While most of the CRISPR/Cas9 based genome editing is reported in seed crops, recently it is also reported in vegetatively propagated crops like banana, cassava and potato (Butler et al., 2016; Odipio et al., 2017; Kaur et al., 2017; Naim et al., 2018; Ntui et al., 2019). Genome editing provides enormous opportunities for improvement of economically important traits of, polyploid, heterozygous and vegetatively propagated crops such as banana and cassava. Illustrative examples are summarized below.

Banana and cassava are among the important staple food and income generating crops for resource-poor farmers in Africa. Their contributions in the African diet are comparable to wheat, rice, maize, or potatoes in other parts of the world. The banana and plantain grown in Africa, is a starchy staple and the major source of carbohydrates to millions of poor people. Similarly, cassava is the most important primary food staple in several African countries. However, there are important biotic and abiotic constraints shattering the production of these important crops in Africa. Therefore, improvement of banana and cassava for economically important traits is critical to fulfill the food demand.

### Tackling Banana Diseases Through CRISPR

Recently, CRISPR-based genome editing of banana has been demonstrated through knocking out the marker gene phytoene desaturase (PDS) leading to albino phenotype, however, the achieved mutation efficiency of 59% was quite low for practical application (Kaur et al., 2017). Further, higher efficiency (100%) of genome editing was reported for dessert banana with using CRIPSR construct with polycistronic gRNAs targeting PDS gene (Naim et al., 2018). Similarly, high mutation efficiency was also reported by IITA using PDS gene as target (Ntui et al., 2019; Tripathi L. et al., 2019). Once the efficient protocol for CRISPR/Cas9-based genome editing was established, this technology was utilized to inactivate the endogenous Banana streak virus (eBSV) integrated in the host genome, overcoming a major challenge in banana breeding (Tripathi J.N. et al., 2019).

BSV is a prevalent virus pathogen developing disease symptoms as chlorotic streaks on leaves and advancement of disease leads to killing of the plant. BSV belong to pararetroviruses, integrated into the host genome and known as endogenous BSV (eBSV). The viral sequences of eBSV are integrated in the B genome derived from Musa balbisiana. Cultivated varieties of banana are polyploid (AA, BB, AAA, AAB, ABB) descended from wild progenitors *Musa accuminata* (A genome) or/and *Musa balbisiana* (B genome). Plantain (AAB), one of the economically important sub-groups of banana, contains one B genome. It is an important staple food crop in Africa. During BSV infection, multiple copies of eBSV sequences integrates at a single locus in the B genome of the host as direct, inverted and tandem repeats (Chabannes et al., 2013). These proviruses can be reactivated into the infectious episomal BSV under several environmental stress conditions. When the infected banana plants are stressed, a functional episomal BSV genome and infectious viral particles are created through recombination of integrated sequences of eBSV, leading to development of disease symptoms in plants. Micropropagation for multiplication of plants through tissue culture and conventional breeding may also trigger activation of eBSV. Hence, the main cause of major epidemics of BSV is not the natural transmission of virus through insect vectors or use of infected seed materials, but instead due to reactivation of integrated eBSV under unfavorable stress conditions. As a result, BSV has become one of the key constraints in genetic improvement of plantain through conventional breeding and also deployment of plantain hybrids.

The diploid progenitor *Musa balbisiana* (BB) or several genotypes with at least one B genome have tolerance to biotic and abiotic stresses and good agronomic traits, still cannot be used as parents in breeding programs. Tripathi J.N. et al. (2019) demonstrated that the endogenous eBSV can be inactivated through targeted knockout of viral sequences from the host plant genome through CRISPR/Cas9 based genome editing and reports a strategy for inactivation of even other endogenous viral genomes from the host plants. The genome-edited events of plantain ‘Gonja Manjaya' with targeted mutations in the viral genome prevented proper transcription or/and translational into infectious viral proteins. The inactivation of eBSV into infectious viral particles was confirmed by testing the potted plants of these edited events under water stress conditions in the glasshouse. Seventy five percent of the tested plants remained asymptomatic under stress conditions, whereas all the non-edited control plants showed BSV disease symptoma. This is the first report of generation of genome-edited crop in Africa and lay the foundation for editing of banana for important traits such as disease resistance. The International Institute for Tropical Agriculture (IITA, Nigeria) is also developing banana varieties resistant to bacterial wilt and fusarium wilt diseases using CRISPR/Cas9 technology.

### Improving Cassava Virus Resistance and Quality

Genome editing of cassava was established using the CRISPR/Cas9 technology for knocking out the Phytoene desaturase (MePDS) gene (Odipio et al., 2017). This technology was further utilized for developing cassava varieties with enhance resistance to the cassava brown streak disease (CBSD), caused by two species of Ipomovirus: Cassava brown streak virus (CBSV) and Ugandan cassava brown streak virus (UCBSV) (Gomez et al., 2018). CBSD is a major viral disease of cassava affecting its production in Central and East Africa. Gomez et al. (2018) reported that targeted mutations in cassava translation initiation factor 4E (eIF4E) isoforms nCBP-1 and nCBP-2 reduces CBSD disease severity as demonstrated with low degree of disease symptoms and virus accumulation in storage tuberous roots upon glasshouse challenge of edited cassava lines with CBSV. Simultaneous mutations in the nCBP-1 and nCBP-2 genes conferred significantly higher resistance to CBSD, however complete resistance to CBSD was not obtained in this study. Therefore, authors recommended that improved varieties of cassava with complete resistance to CBSD can be developed by stacking this approach of disrupting eIF4E isoforms with other resistance strategy such as RNAi.

Further, researchers has attempted to apply this technology to develop enhance resistance to African cassava mosaic virus (ACMV), a geminivirus (Mehta et al., 2019). However, the effective resistance to ACMV was not achieved in the glasshouse challenge experiments. The authors linked this to the evolution of editing-resistant geminiviruses in edited cassava.

CRISPR/Cas9-based genome editing can be coupled to genetic improvements in cassava for traits such as starch improvement and early flowering. Bull et al. (2018) reported genome-editing of cassava for manipulating starch biosynthesis and improving the starch quality in the storage roots. They generated the edited cassava with mutations in two genes [PROTEIN TARGETING TO STARCH (PTST1) or GRANULE BOUND STARCH SYNTHASE (GBSS)] involved in amylose biosynthesis, leading to reduction or elimination of amylose content and finally improving the quality of in starch in cassava roots. The authors also demonstrated accelerated breeding by transferring Arabidopsis FLOWERING LOCUS T gene in the genome-editing events of cassava for early flowering. They further demonstrated edited cassava with modified starch can be segregated in greenhouse to produce transgene-free progeny with improved trait.

In order to meet the increasing demand of food with limited or same resources, better and effective ways to produce food are required. As summarized above, one option is to utilize new breeding tools like genome editing for crop improvement. Currently, severe endeavors are underway to enhance yields of banana and cassava—among a range of other crops—through generating improved varieties with resistance/tolerance to biotic stresses.

A major question to be addressed, from a regulatory perspective, will the products of NBTs be classified under the current definitions of genetic modification, or not? Over the last three decades, a patchwork of (draft) biosafety laws and regulations has emerged affecting the development and release of improved crops and resulting in trade issues when approvals are “asynchronous”^4^. In recent years, given the rapid advances in genome editing, there is increased regulatory attention and debate around these applications. Salient developments are outlined below; it should be emphasized that while an increasing number of regulatory authorities have provided clarity regarding their approach to genome edited crops, the jury is still out in major economic blocs such as the European Union.

## Emerging Regulatory Approaches to Genome Editing, With Reference to Africa

Considering the above sketched situation, where differences in GMO regulation are exacerbated by specific challenges posed by NBTs, different countries are responding in different ways to the question of how applications of NBTs should be classified, as regulated GM material or not. The key point here is that specific applications, e.g., when genome editing is used to create a loss of function of a target gene, result in an event that contains no “foreign DNA”, i.e., no novel combination of genetic material and therefore cannot be distinguished from a product of conventional mutagenesis (which is commonly exempt from GM regulation).

### Summary of Global Developments

As early as 2013, the European Academies Science Advisory Council (EASAC), a body of national science academies of the EU Member States, argued that products of NBTs should not fall under GMO legislation when they do not contain “foreign DNA”. The EASAC advisors noted that in some cases the product cannot be distinguished from one generated by conventional techniques, and also argued that the new techniques allow much more precise and targeted changes compared with mutagenesis used in conventional breeding, where changes in the genome are induced by chemicals or radiation, creating multiple, unknown, and unintended mutations (EASAC, 2013).

Regulatory authorities in a range of countries follow this same EASAC conclusion that genome editing, in cases where no novel combinations of genetic material have been created, should be no more regulated than a product of conventional mutagenesis. An often-cited case in point is Argentina, where in 2015 a new regulation was issued aimed at clarifying the regulatory status of products from NBTs. The regulation allows applicants to consult with the competent authority early-on in the R&D stage (“design stage”) to determine if a product developed using gene editing is a GMO or not. The prime decision-making factor here is if the product has a *novel combination of genetic material* or not. Preliminary consultations are then followed by a final determination based on a full information package describing the gene editing procedure and resulting changes in the genomic sequences of the end product (Lema, 2019). So far, this approach has resulted in regulatory exemptions for several genome edited crops in Argentina. Importantly, South American countries such as Brazil, Chile, Colombia and Paraguay have followed Argentina's lead and will regulate genome edited products on a case-by-case basis and allow exemptions from GM regulation when there is no novel combination of genetic material. Similar approaches can be cited from major economies such as the USA, Japan and Australia. Such decisions regarding NBTs generally followed a thorough review of scientific evidence and existing regulations so that these remain fit for purpose in times of rapid technological development.

By contrast, in a major agricultural trading bloc like the EU there is still uncertainty as to how products from NBTs will be regulated. While EU bodies recognized the potential of NBTs early on, as evidenced by a range of studies and projects conducted with EU support, to date there has been no clear-cut policy decision or statement from the European Commission. So far its actions were limited to, among other things, requesting advisory notes from the EU Scientific Advisory Mechanism (SAM) and awaiting a legal opinion from the EU Court of Justice (CJEU). This wait-and-see approach has greatly complicated matters.

On 25 July 2018, the CJEU advised that organisms obtained by new mutagenesis techniques are considered as GMOs, within the meaning of the EU's Directive 2001/18/EC on the release of GMOs into the environment (the “GMO Directive”), and that they are subject to the obligations laid down by the GMO Directive (Curia Press and Information, 2018). Thus, the ruling considers genome edited organisms as GMOs, which do not fall under the existing exemption under the Directive for organisms resulting from conventional mutagenesis. The ruling adopts a strict legal interpretation of what constitutes a GMO and does not consider the principle of “novel combination of genetic material” as applied in other jurisdictions – as summarized above. It should be noted that the Court ignored this principle while the GMO Directive includes, in its definition of a GMO, the phrase *“has been altered in a way that does not occur naturally by mating and or natural recombination”*. This misunderstanding implies that organisms that have been edited but that can or do occur naturally, will have to follow the same European regulatory procedures as GMOs, including a detailed analysis of possible risks.

The Court ruling has been widely debated since its publication, which will not be summarized here. A clear analysis of its implications was issued by the European Commission's Group of Scientific Advisors (2018). The Group concludes that:

*“[ … ] meeting the obligations of the GMO Directive implies cost- and labour-intensive pre-market evaluations and a long duration of the approval process, which are difficult and onerous to bear, particularly by small and medium enterprises. This may diminish incentives for investment, negatively affect research and innovation in this field, and limit the commercialisation of gene edited products.”* (EC-SAM, 2018)

In particular, it is noted that:

*“It is a concern that countries in the developing world exporting feed and food to the EU might not benefit from gene edited crops if they follow the EU authorisation practices, as some of them currently do. No single breeding technique alone can provide a magic bullet for solving the problem of unsustainable food production and food scarcity in the world. However, gene- editing has the potential to contribute to food security, which is particularly relevant given the growing world population and climate change.”* (EC-SAM, 2018)

A critical challenge, also emphasized by EC-SAM (2018), to the EU decision-making process will be the fact that enforcement of obligations imposed by the GMO Directive, on traceability and labeling of GMOs entering the EU will be near-impossible. Due to the absence of a novel combination of genetic material, seeds and commodities developed with NBTs are identical to those developed through unregulated plant breeding or naturally occurring variations. As a result, the detection, identification and quantification of genome edited products will be a major challenge. This fact will become more problematic when exporting countries authorize the cultivation of genome edited crops that will not be regulated as GMOs.

In order to address this situation, EC-SAM recommends amending the EU's GMO Directive to reflect the growing track record of safe use and consumption and associated scientific evidence, in particular on genome editing and established techniques of genetic modification, considering the obligation for GMO legislation to be: *“[C]lear, evidence-based, implementable, proportionate and flexible enough to cope with future advances in science and technology in this area”* (EC-SAM, 2018).

### Emerging Regulatory Approaches in Sub-Sahara Africa

Considering the situation sketched above, and the important influence the EU has in shaping regulatory policies in Africa due to trade relations and historical ties, it is encouraging to note that sub-Sahara African governments have started defining their own regulatory approaches to GMOs and, increasingly, applications of NBTs. Based on growing expertise worldwide and in-country with safety reviews and decision-making on GMOs, including general releases of GM insect-resistant cotton and cowpea, regulatory authorities are now getting ready for NBTs and genome editing. Apart from global regulatory developments in other parts of the world, there are several important drivers behind regulatory policy formulation in Africa, including:

1. Rapid technological developments resulting in the first contained-use applications involving NBTs being recently submitted to regulatory authorities in Africa, for example, in Kenya by international research centers.
2. Discussions as part of the CBD's bi-annual inter-governmental meetings regarding synthetic biology, gene drives, genome editing, among other items, which often take a highly precautionary view on emerging technologies generally and NBTs in particular, including the calling for moratoriums (Callaway, 2016), which were refuted by a majority of African delegates.
3. Policy consultations under the umbrella of the African Union, proposing more enabling and science-based approaches to emerging technologies such as gene drives and genome editing. While the Union's work on genome editing started only recently, its report on gene drives clearly embraces the technology as a realistic option for effective disease control (AU-NEPAD, 2018).

Spurred by these developments, a few governments are considering the inclusion of NBTs and other emerging technologies in their regulatory frameworks. A case in point is Nigeria, where, in August 2019, an amended biosafety act for Nigeria was published in the government gazette following assent by the country's President. The amendments broaden the scope of the act to wider “biosecurity” concerns, not just biosafety, and to include applications of genome editing, gene drives and synthetic biology as regulated technologies along with GMOs. At this stage, no specific assessment criteria and procedures were defined, nor criteria for possible exemptions. This approach may be effective as it brings the new tools and technologies under the purview of a functional regulatory agency; however, it may open the door for regulating genome editing innovations as if they are GMOs. Further work will be undertaken by Nigeria's National Biosafety Management Agency (NBMA), which would lead to a science-based guideline that would help implement the amended Act by including clear regulatory triggers for genome edited organisms.

Rather than amending its biosafety act, in Kenya the National Biosafety Authority (NBA) opted to develop a guideline on genome editing. Guideline development was deemed to be a suitable approach as it allows for flexibility, in a rapidly evolving field, and consultations with scientists and regulatory agencies in the agricultural and environmental sectors. Following initial consultations, NBA organized a technical meeting in April 2019 analyzing advances in genome editing, and relevant regulations in Kenya as well as other countries. Currently, the authority is drafting a guideline that aspires to be practical and science-based, and which allows for case-by-case reviews and exemptions from biosafety review for products that do not have a novel genetic combination. Thus, the proposed approach for Kenya essentially follows those adopted in Argentina and other South American countries.

It is expected that other countries that embrace agricultural biotechnology will soon follow Nigeria's and Kenya's lead and devise policy and regulatory approaches to NBTs and other emerging technologies.

## Way Forward and Recommendations

In this article, we have pointed to several important recent developments in sub-Sahara Africa regarding the regulation and adoption of GM crops, as well as the continuing political challenges that hamper further progress. Regulatory authorities in select case study countries have gone through a period of rapid capacity development, as shown by the increasing number and scope of GMO environmental releases including CFTs and general releases and have shown flexibility in their decision-making processes. A major driving factor in this process constitute the more progressive agricultural and development policies as formulated by national governments. Recent capacity development will provide a foundation for the formulation and implementation of science-based regulations for novel breeding techniques such as genome editing.

While recent progress is encouraging, we fully acknowledge the fact that political challenges remain. A critical challenge involves the need to sustain the political will and current momentum that provides scientists with leeway to operate. While not discussed extensively in this article, it is generally recognized that controversies exist around the adoption of GM crops and that, despite a safety track record of over two decades, public opinion on this topic remains divided. These controversies are sometimes reflected in government decisions in sub-Sahara Africa, when decision-makers call for moratoriums on CFTs or bans on GM commodity imports. Such calls are influenced by activist groups who campaign against modern agricultural technologies generally, and against GMOs in particular. Recently, these campaigns have also resulted in court cases challenging biosafety decisions by national competent authorities. Despite this, progress in Africa continues but it involves a careful balancing act.

Governments and development partners will have to continue investing in the development of knowledge, skills and capacities required to regulate and adopt GM crops; and, in due course, the products emanating from genome editing applications—as introduced in this article. Emerging best practices from, e.g., Argentina, provide critical guidance. Capacity development should include outreach and awareness initiatives to ensure public debates and policy consultations are well informed and incorporating the best available science.

A special case is made here for enhanced regional and sub-regional collaboration. Increasingly, regulatory authorities in Africa are exchanging expertise and data regarding biosafety decisions, including the acceptance of data generated in neighboring countries. This collaboration would provide the basis for harmonization efforts in regional economic communities such as the Common Market for East and Southern Africa (COMESA), the Economic Community of West African States (ECOWAS) and the Southern African Development Community (SADC). Each of these regional bodies have initiated processes and guideline development towards sub-regional harmonization in the recent past but none have been adopted and implemented so far. A constructive development at the regional level involves the recent policy statements by the African Union (AU) regarding genome editing and gene drives for human health purposes, which have impacted discussions in AU member states. Such supportive statements will continue to be important to bolster the current momentum.

## Data Availability Statement

All datasets generated for this study are included in the article/supplementary material. 

## Author Contributions

All authors listed have made substantial, direct, and intellectual contribution to the work and approved it for publication.

## Conflict of Interest

Author JK was employed by the company Komen Consultancy.

The remaining authors declare that the research was conducted in the absence of any commercial or financial relationships that could be construed as a potential conflict of interest.

## Footnotes

1 Following World Bank (2011), this article uses a broad depiction of ‘innovation', encompassing technological change and institutional change. Following World Bank's agricultural innovation systems sourcebook (2011), components of innovation include, in addition to a strong capacity in R&D, collective action and coordination, the exchange of knowledge among diverse actors, the skills, incentives and resources available to form partnerships and develop businesses, and enabling conditions that make it possible for actors to innovate.

2 DUS/VCU, Distinctness, Uniformity and Stability/Value for Cultivation and Use.

3 It is beyond the scope of this article to provide a detailed introduction on NBTs. For an excellent introduction, refer to the series of factsheets published by the European Plant Science Organisation, EPSO (2016). URL: https://epsoweb.org/epso/fact-sheets-on-new-breeding-technologies/2016/03/21/

4 Asynchronous approval refers to the situation in which there is a delay in the moment when a GM event is allowed to be used in one country in comparison to another country. A notable case in point illustrating the trade disruptions from asynchronous GMO approvals concern the use of GM plant varieties that are approved in countries which export them to the EU, mainly in the form of animal feed (maize, soybeans), before these are actually approved by the EU.
